# Systematic epistatic mapping of cellular processes

**DOI:** 10.1186/s13008-016-0028-z

**Published:** 2017-01-06

**Authors:** Maximilian Billmann, Michael Boutros

**Affiliations:** 1 German Cancer Research Center (DKFZ), Division Signaling and Functional Genomics and Heidelberg University, Department of Cell and Molecular Biology, Faculty of Medicine Mannheim, Im Neuenheimer Feld 580, 69120 Heidelberg, Germany; 2 German Cancer Consortium (DKTK), 69120 Heidelberg, Germany; 3 Department of Computer Science and Engineering, University of Minnesota-Twin Cities, 200 Union St SE, Minneapolis, MN 55455 USA

**Keywords:** Genetic interactions, Image analysis, RNAi, Cell cycle

## Abstract

Genetic screens have identified many novel components of various biological processes, such as components required for cell cycle and cell division. While forward genetic screens typically generate unstructured ‘hit’ lists, genetic interaction mapping approaches can identify functional relations in a systematic fashion. Here, we discuss a recent study by our group demonstrating a two-step approach to first screen for regulators of the mitotic cell cycle, and subsequently guide hypothesis generation by using genetic interaction analysis. The screen used a high-content microscopy assay and automated image analysis to capture defects during mitotic progression and cytokinesis. Genetic interaction networks derived from process-specific features generate a snapshot of functional gene relations in those processes, which follow a temporal order during the cell cycle. This complements a recently published approach, which inferred directional genetic interactions reconstructing hierarchical relationships between genes across different phases during mitotic progression. In conclusion, this strategy leverages unbiased, genome-wide, yet highly sensitive and process-focused functional screening in cells.

## Background

**Fig. 1:**
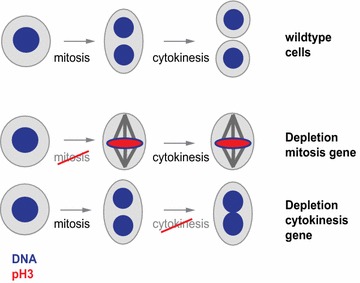
Schematic illustration of a cell progressing through mitosis and cytokinesis. Mitotic arrest or a cytokinesis defect can be introduced by depleting genes involved in mitotic progression or cytokinesis and cause visual changes that can be measured using markers for mitotic chromosomes (*red*) or total DNA (*blue*)

During cell division, a cell undergoes several consecutive events to replicate and divide its genome and distribute it to two daughter cells. The temporal order and mechanism of those events has been extensively explored using methods that visualize the DNA content or size of cells [1] or their content of cell cycle-specific proteins such as cyclins [2]. Microscopy techniques illustrate the localization of cellular components [3–5] and cell cycle specific factors or the presence of cell cycle markers such as residue-specific phosphorylation of histone H3 [6, 7]. Image analysis algorithms can capture such cellular structures in an automated fashion. For example, this enables quantification of the fraction of cells with condensed chromosomes or visible serine 10 phosphorylated histone H3 (pH3) levels as a proxy for cell cycle defects. Importantly, image analysis can extract multiple phenotypic features from a cell population, which allows for simultaneously following distinct biological processes. For instance, automated analysis of cells stained for their DNA and pH3 visualizes perturbations causing defects in mitotic progression (increased mitotic index in the cell population) or cytokinesis (increased average nuclear size) (Fig. 1).

To identify regulators of the cell cycle in a systematic fashion, model systems such as budding yeast, cultured *Drosophila* or human cells have been exploited in genome-scale functional screens [8–10]. Advanced automated image analysis have enabled screening for modulators of diverse biological processes [11–13]. For instance, an RNAi screen with live imaging of human HeLa cells has exploited a stably expressed GFP-labeled histone H2B to identify genes required for proper chromosome segregation and cell cycle propagation [13]. While such studies have identified cell cycle regulators with high sensitivity, genetic interaction analysis approaches have been able to define functional and epistatic relations between genes [14]. Genetic interaction analysis systematically exploits genetic buffering by genetic variants, which completely or partially overlap in function [14–17]. Genetic interaction studies have been performed in yeast and assayed cell fitness as a composite phenotype [14, 16] capturing a broad spectrum of biological processes such as sister chromatid segregation, cytokinesis or the mitotic exit [18–20]. Such genetic interaction analyses have successfully been applied to further characterize hits from single gene screens [19, 21]. Recently, genetic interaction analyses approach in yeast have increased the throughput to the genome-scale [22] and reported a close-to-complete coverage of all gene pairs by measuring ~23 × 10^6^ combinatorial knockouts [23]. To score genetic interactions in a metazoan model system, we developed an approach that uses systematic combinatorial RNAi in cultured *Drosophila* cells [24, 25].

Here, we discuss a recent study by our laboratory, which focuses on cell cycle-relevant phenotypic features in *Drosophila* cells and uses genetic interaction mapping to visualize functional networks underlying mitotic progression and cytokinesis [26]. This study characterized novel modulators by genome-wide high-content imaging RNAi screening, and structured the resulting ‘hit’ list using mitotic index- and nuclear area-focused genetic interaction analysis.

## Discussion

### Distinct phenotypic features guide the detection of specific genetic interactions

**Fig. 2:**
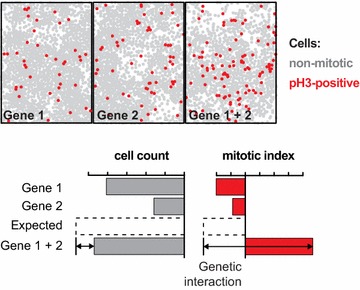
Genetic interactions specifically affect distinct phenotypic features. Example showing that the image-derived feature cell count did not reconstruct the genetic interaction, but the fraction of mitotic cells (mitotic index) provided this information. Figure was modified from [33]

Cultured *Drosophila* cells have been used for genome-scale loss-of function screens [9, 27–32] many of which investigating regulators of the cell cycle [9, 30–32]. Billmann and colleagues have screened the genome for cell cycle regulators by acquiring multiple phenotypic features through high-content imaging [26]. The comparison between cell count as a surrogate for fitness with the mitotic index of the population or the average nuclear area showed that the latter two features identified additional hits [26]. We selected roughly 300 genes affecting the mitotic index and nuclear size. Using genetic interaction analysis, networks were generated connecting genes that showed epistatic similarity when the mitotic index or nuclear area was measured. Often, genetic interactions were detected in the mitotic index but not when considering cell fitness only, thus providing additional, process-specific information for network generation (Fig. 2). Here, we discuss two analysis strategies that exploit this observation. Non-redundant multi-feature epistatic information enables inferring temporal functional relations between genes, while feature-specific epistatic profiles can reconstruct process-specific functional networks (Fig. 2).

### Multi-phenotype interactions can reconstruct directed hierarchies

**Fig. 3:**
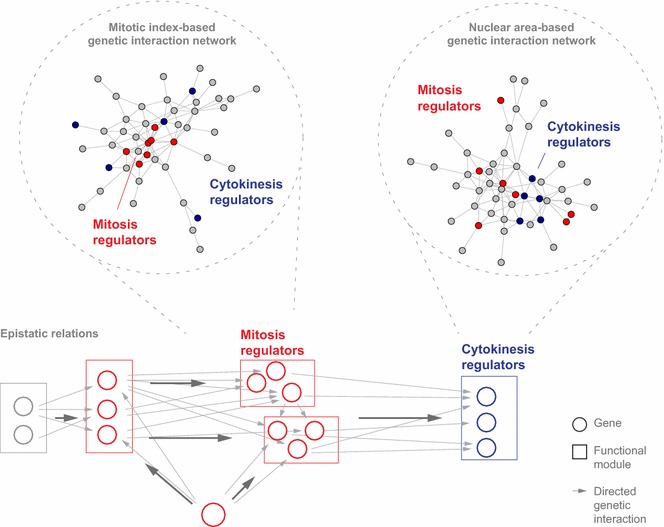
Temporal resolution of genetic interaction networks and genome-scale process-specific functional association of gene function. The epistatic relations between functional modules are reconstructed based on directed genetic interactions inferred from multi-feature genetic interaction profiles. The networks present functional relations as inferred from correlations between process-specific, single feature-focused genetic interaction profiles

Recent work from our laboratory has identified epistatic relationships between genes building networks reflecting temporal order of gene function in processes such as the mitotic cell cycle [33]. Epistatic relationships were reconstructed by directed genetic interactions, indicating whether one gene repressed or amplified another genes effect (Fig. 3). This direction was inferred by comparing multi-feature phenotypic profiles of two genetically interacting genes with their combinatorial knockdown profile [33]. Phenotypic features were taken from cells stained for their DNA content, the cytoskeletal component beta-tubulin as well as for the presence of pH3, and described population features as well as the shape and texture of cells [33].

For instance, this reconstructed an epistasis network between the components of functional modules of the mitotic cell cycle comprising structural modules such as the γ-tubulin ring complex, Condensin or Cohesin, regulatory modules such as the anaphase-promoting complex/cyclosome (APC/C) or the spindle assembly checkpoint (SAC), motor proteins (Dynein, Dynactin) and regulatory genes such as *polo* (*Drosophila PLK1*) [33]. This approach demonstrated that multi-parametric genetic interaction-based networks associate gene function and, in addition, provide epistatic relationships, thereby systematically visualizing functional relations between genes. Finally, features derived from the mitosis marker pH3 were highly informative for functional modules regulating mitosis, suggesting that feature-specific genetic interaction networks provide a snapshot of functional relations in the specific biological process.

### Phenotype-specific genetic interactions visualize process-specific networks

**Fig. 4:**
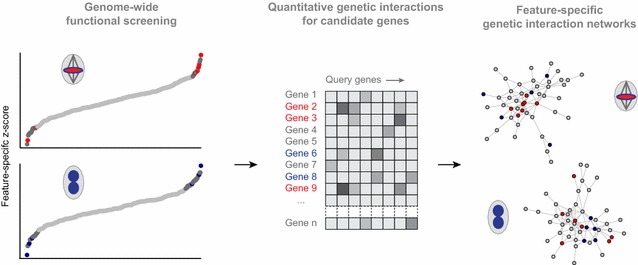
Two-step screening approach to prioritize candidate genes from multi-parametric genome-wide functional screens (*left*), and functionally relate them by genetic interaction analysis (*middle*, *right*)

To build functional networks of distinct cell cycle phases by genetic interaction analysis, we used high-content imaging of cell cycle features and a two-step screening approach: first, we screened a genome-wide RNAi library [34], measured genetic interaction profiles of selected genes and used this data to infer functional similarity [26] (Fig. 4). The genome-wide screen followed the rational that genes with a more pronounced depletion phenotype tend to genetically interact with a large fraction of genes [33, 35]. The authors added genes with moderate phenotypic strength. To generate feature-specific genetic interaction networks, they used the mitotic index, the fraction of pH3-positive cells in the cell population, which serves as a proxy for mitotic progression. Mitotic index-based genetic interactions reconstructed regulatory modules of mitosis, but failed to group known components required for cytokinesis [26]. Clustering the latter components required the phenotypic feature nuclear size. This feature captures large, multi-nucleated cells, which arise due to endoreduplication after failed cytokinesis [36]. Together, this approach generated genome-scale functional networks for specific cell cycle phases.

Those networks functionally assigned many potentially novel cell cycle regulators, which had often been described in processes not directly connected to cell cycle regulation. For those genes, their phenotypic strength alone insufficiently guided hypothesis generation. The second-line genetic interaction mapping approach deprioritized many of those hits, while suggesting hypothesis for others such as Golgi-resident components during mitotic progression [26].

## Conclusions

Multi-feature imaging enables the visualization of epistatic relationships between genes by considering genetic interactions along the vector of phenotypic features such as cell count, mitotic index and nuclear area [5, 33]. Moreover, genetic interactions affecting one process-specific feature capture a network of functional relations, zooming into a step of the causal chain in biological processes (Fig. 3).

Methodological rapid advances in CRISPR-based screens in mammalian systems and small molecule screens will require robust experimental and computational strategies to guide testable hypothesis. For example, a recent study in yeast generated various distinct phenotypic reporters by endogenously tagging various proteins with a GFP. The authors subsequently applied deep learning algorithms to the images to define cellular compartments and assess the response to genetic perturbations at multiple phenotypic levels [37]. Recently, a method integrated this high-content approach with a technique for systematic genetic interaction analysis in yeast [38], which will enable building networks illustrating functional relations in various biological processes.

Recent studies have also shown how to use image-based screening for cellular phenotypes after treatment with small molecules in different genetic backgrounds to functionally group ~1300 pharmacologically active compounds [39]. Two-step screening approaches would allow to extent the number of screened small molecules by several orders of magnitude, while sensitively mapping the mode of action for pre-selected compounds.

Mapping gene function using genetic interactions has also been performed in mammalian cells [40–42]. Due to the larger genome size, two-step genetic interaction screening approaches provide an attractive strategy. While combinational RNAi face several challenges [43], more recent gene editing CRISPR/Cas9-based technologies enabled efficient and reliable gene perturbation across human cells [44]. In combination with a scRNA-seq phenotypic readout, pooled CRISPR screens can be exploited to build process-focused genetic interaction networks in higher organisms [45]. Eventually, multi-step combinatorial gene depletion approaches will help building a systems view of biological processes such as the cell cycle [46] across genetic model systems.

## Abbreviations

RNAi: RNA interference
pH3: phosphorylated histone H3
APC/C: anaphase-promoting complex/cyclosome
SAC: spindle assembly checkpoint
GFP: green fluorescent protein
CRISPR: clustered regularly interspaced short palindromic repeats
scRNA-seq: single cell RNA sequencing
